# Dynamics of Serum Pregenome RNA in Chronic Hepatitis B Patients Receiving 96-Month Nucleos(t)ide Analog Therapy

**DOI:** 10.3389/fmed.2022.787770

**Published:** 2022-02-28

**Authors:** Yachao Tao, Menglan Wang, Juan Liao, Xing Cheng, Min He, Dongmei Zhang, Taoyou Zhou, Jie Chen, Enqiang Chen, Hong Tang

**Affiliations:** ^1^Center of Infectious Diseases, West China Hospital, Sichuan University, Chengdu, China; ^2^Division of Infectious Diseases, State Key Laboratory of Biotherapy, Sichuan University, Chengdu, China; ^3^Shanghai RenDu Biotechnology Co. Ltd, Shanghai, China

**Keywords:** pregenome RNA, chronic hepatitis B, HBV DNA, hepatitis B e antigen seroconversion, nucleos(t)ide analogs

## Abstract

**Background:**

Tissue covalently closed circular DNA (cccDNA) can reflect the activity of HBV replication. However, it is impractical to assess intrahepatic cccDNA in every outpatient. Serum pregenome RNA (pgRNA) is transcribed from intrahepatic cccDNA and may reflect the activity of intrahepatic cccDNA. We explored the dynamics and the potential role of serum pgRNA in patients receiving long-term NAs treatment.

**Methods:**

Serum pgRNA, HBV DNA, HBsAg, HBeAg, and ALT levels were quantified, and the relationships between serum pgRNA and these common clinical indicators before and after the treatment were investigated.

**Results:**

Serum pgRNA showed dynamic change during the 96-month NAs therapy, and serum pgRNA levels were positive and detectable in 19 patients with undetectable serum HBV DNA. Serum pgRNA showed strong and positive correlation with serum HBV DNA (*r* = 0.693, *p* < 0.001) and serum HBsAg levels (*r* = 0.621, *p* < 0.001) at baseline. Patients with HBeAg seroconversion had lower baseline serum pgRNA levels (*p* = 0.002). The area under the curve (AUC) of baseline serum pgRNA for predicting HBeAg seroconversion was 0.742 (95% CI: 0.606–0.850) with 63.16% sensitivity and 80.56% specificity. The cumulative HBeAg seroconversion rate was higher in patients with low serum pgRNA (*p* = 0.001).

**Conclusion:**

Serum pgRNA of low level at baseline or great decline at month 6 may independently predict the high incidence of undetectable serum pgRNA at year 4 following NAs therapy, and the baseline serum pgRNA may serve as a novel predictor for HBeAg seroconversion during NAs therapy.

## Introduction

Hepatitis B virus (HBV) infection remains one of the main challenges to human health, and chronically infected patients are at increased risk of developing liver cirrhosis and hepatocellular carcinoma (HCC). Current standard of care treatments against chronic HBV infection using nucleos(t)ide analogs (NAs) and/or pegylated- interferon can effectively block reverse transcription of HBV and suppress viral replication (1). They are aimed at decreasing the risk of HBV-related severe complications and improving the survival rate.

HBV is defined as a partially double-stranded DNA virus that replicates and assembles exclusively in hepatocytes. Once HBV enters human hepatocytes, the viral particle releases nucleocapsid, and the genome-containing nucleocapsid transports to the nucleus to release the relaxed circular DNA (rcDNA). The viral genome contains four major open reading frames (ORFs), namely C, P, S, and X, leading to the synthesis of several functional proteins, including the HBc and HBe antigen, viral polymerase, three envelope proteins and the regulatory protein HBV X (HBx). The rcDNA is converted to covalently closed circular DNA (cccDNA) which yields viral RNA of different lengths: including 3.5-kb precore RNA and pregenomic RNA (pgRNA), 2.4- and 2.1-kb RNA producing surface proteins, and a 0.7-kb RNA encoding HBx protein (2). After nuclear export, the HBV pgRNA is translated into viral polymerase and core proteins, providing for the self-assembly of the RNA-containing viral nucleocapsid. And the immature RNA-containing viral nucleocapsids are converted to the DNA containing nucleocapsids by pgDNA reverse transcription. The DNA-containing nucleocapsids interact with viral surface proteins for the formation of mature viral particles, which are then released from hepatocytes (3).

The goal of serum HBsAg loss with or without seroconversion, or termed functional cure, is the ideal endpoint of current treatment strategies. However, the number of patients achieving functional cure is far from satisfactory using current antiviral therapeutics. Most patients require long-term antiviral therapy, and viral relapse is common after cessation of therapy owing to the persistence of cccDNA in the infected hepatocytes. The reduction of intrahepatic cccDNA was minimal although dramatic reduction in serum HBV DNA level was observed after 1 year of NAs therapy, and cccDNA was still detected in patients with undetectable serum HBV DNA (4). Thus, serum HBV DNA cannot accurately reflect intrahepatic cccDNA activity. Quantifying intrahepatic cccDNA activity may be an ideal surrogate marker in evaluating the antiviral efficacy and treatment endpoint for CHB patients, but it is impractical to regularly assess intrahepatic cccDNA in every outpatient. Thus, non-invasive biomarkers indicating intrahepatic cccDNA activity and monitoring therapeutic effects are being explored.

Serum HBsAg quantification has been reported to correlate well with tissue cccDNA (5). Among patients who stopped antiviral therapy according to the recommendations of the Asian Pacific Association for the Study of the Liver (APASL), the 1-year cumulative ratio of viral rebound was 42% in HBeAg-positive patients and 47% in HBeAg-negative patients, and serum HBsAg levels were lower in patients who did not develop viral relapse (6). HBsAg loss/seroconversion represents the safest current treatment endpoint, but it is a rare event during NAs therapy. Moreover, HBsAg titer cannot completely represent hepatic cccDNA activity and a decline in serum HBsAg does not correlate well with a reduction in intrahepatic cccDNA, because HBsAg has two sources: cccDNA and integrated HBV DNA. Current commercially available HBsAg quantitative assays detect all forms of circulating HBsAg and cannot distinguish where HBsAg proteins come from (7). Besides, when HBsAg escape mutants are predominant, HBsAg quantitation may be inaccurate (8). Thus, other serum biomarkers, such as pgRNA, have been advocated as novel serum markers to predict the prognosis and treatment response. HBV pgRNA is derived from the cccDNA (9), it may be a potential non-invasive biomarker reflecting intrahepatic cccDNA activity (10). Recent studies have demonstrated the predictive value of serum pgRNA for treatment response to pegylated-interferon in HBeAg-positive and -negative patients (11, 12).

In the present study, we quantified serum pgRNA, HBV DNA, HBsAg, and HBeAg levels and investigated the relationships between serum pgRNA and these common clinical serological indicators before and after treatment to corroborate whether serum pgRNA is a strong biomarker to monitor the antiviral efficacy in CHB patients receiving long-term NAs treatment.

## Materials and Methods

### Patients

Total of 88 patients were retrospectively screened from our previous published study, in which all individuals started standard dose of NAs between 2007 and 2008 and received at least 8-year antiviral therapy [entecavir (ETV)/ lamivudine (LAM)/ adefovir dipivoxil (ADV)/ telbivudine (LdT)] (13). All these patients were regularly followed up every 3–6 months for clinical assessment during NAs treatment and serum samples were collected every visit time-point from baseline to month 96. The exclusion criteria were as follows: (1) patients co-infected with other viruses; (2) patients with the evidence of autoimmune liver diseases, Wilson's disease, drug-induced liver injury, and significant intake of alcohol; (3) Patients with cirrhosis-related complications, for example, HCC. Verbal informed consent was obtained from all participants.

### Laboratory Measurements

Serum alanine aminotransferase (ALT) level was tested depending on standard procedures (Olympus AU5400, Tokyo, Japan) in the clinical laboratories. HBV genotypes were determined by direct *S*-gene sequencing. The double nucleotide A1762T and G1764A exchange, or termed TA mutant, was detected with commercially available Line Probe Assays (INNOGENET-ICS, Belgium).

Serum HBsAg titer was measured using Elecsys® HBsAg II Quant Assay (Roche Diagnostics, Penzberg, Germany) according to the manufacturer's instructions. HBeAg and anti-HBe were checked by Roche Modular Analytics E170 Assay (Roche Diagnostics, Indianapolis, IN).

Serum HBV DNA level was quantified using Cobas Taqman Assay (Roche Diagnostic Systems, Branchburg, NJ). The threshold of serum HBV DNA is 100 IU/mL in our hospital, and HBV DNA testing results below 100 IU/mL were considered negative.

Serum HBV pgRNA was measured by Simultaneous Amplification and Testing (SAT) method with HBV-SAT kit (Shanghai Rendu Biotechnology Co., Ltd. China), as previously described (14). SAT is a novel nucleic acid detection technology combining isothermal RNA amplification with real-time fluorescence detection. Briefly, RNA was extracted from serum using magnetic microparticles with HBV-specific RNA oligonucleotides following the manufacturer's instructions. The target RNA was reverse transcribed to double strand DNA containing T7 promoter, and the DNA subsequently served as a template for transcription by T7 RNA polymerase. The synthesized RNA combined with beacon probe labeled with fluorescence and was finally detected. The serum HBV pgRNA level was normalized to the internal control (IC) nucleic acid. The linear quantification range of the assay was from 2 to 8 log copies/mL, with 50 copies/mL as the lower limit of detection.

### Definition

Virological response during NAs treatment is defined as undetectable serum HBV DNA level using sensitive polymerase chain reaction (PCR) assay. HBeAg seroconversion is defined as HBeAg loss and seroconversion to anti-HBe in HBeAg-positive patients (1).

### Statistical Analysis

Continuous variables are expressed as mean ± standard deviation or median with interquartile ranges (IQR), and compared using *t-*test or Mann-Whitney *U-*test, as appropriate. Serum HBV DNA, HBsAg titer, and serum pgRNA levels were presented as log transformation. Categorical variables were presented as count and percentage and were compared using chi-squared test. The correlation between continuous variables was tested by Spear-man's bivariate correlation. Univariable and multivariate logistic regression analysis was performed to identify predictors for undetectable level of serum pgRNA at year 4. The receiver operating characteristic curve (ROC) was used to evaluate the validity of serum pgRNA in predicting HBeAg seroconversion. Cumulative HBeAg seroconversion rate was analyzed by the Kaplan-Meier method with a log-rank test. All statistical tests were two-sided and a *p*-value <0.05 was considered significantly different. The statistical software package used was SPSS Version 18.0 for Windows (SPSS, Chicago, IL, USA).

## Results

### Baseline Characteristics

Total of 88 patients with chronic HBV infection were incorporated, including 79 hepatitis patients and nine cirrhotic patients. Among these patients, 19.3% (17/88) presented a serum ALT level <2× upper limit of normal (ULN) and 63.6% (56/88) showed more than 5× ULN ALT level. All patients had either HBV genotype B (67%) or C (33%). Nearly half of the patients (48.9%) were detected with high viral load (>7.7 log10 IU/mL), 51.1% with low or medium viral load. About 61.4% of patients had HBsAg levels ≤ 4 log10 IU/mL and 38.6% had HBsAg levels >4 log10 IU/mL, respectively. The median value of serum pgRNA in all patients was 6.91 log10 IU/mL. The number of patients receiving ETV was equal to that of patients receiving other antiviral drugs (LAM/ADV/LdT). The detailed demographics and laboratory data are shown in Table 1.

**Table 1:** Baseline parameters of all 88 patients.

**Parameter**	**Value**
Age starting antiviral therapy, years	35 (17–57)^a^
<40	60 (68.2)^b^
≥40	28 (31.8)^b^
Male sex	63 (71.6)^b^
BMI, kg/m^2^	21.99 (16.90–31.62)^a^
Family history of hepatitis B	49 (55.7)^b^
Cirrhosis	9 (10.2)^b^
ALT, IU/mL (×ULN)	2.73 (1.04–9.96)^a^
<2 × ULN^*^	17 (19.3)^b^
2~5 × ULN	56 (63.6)^b^
≥5 × ULN	15 (17.0)^b^
HBV genotype	
B/C	59 (67.0)/29 (33.0)^b^
TA mutant	31 (35.2)^b^
HBeAg-positive	55 (62.5)^b^
HBV DNA, log10 IU/mL	7.69 (2.97–10.09)^a^
Low/Medium ( ≤ 7.7)	45 (51.1)^b^
High (>7.7)	43 (48.9)^b^
HBsAg, log10 IU/mL	3.70 (2.35–5.26)^a^
≤ 4	54 (61.4)^b^
>4	34 (38.6)^b^
pgRNA, log10 IU/mL	6.91 (3.35–9.30)^a^
Antiviral treatment	
ETV/other (LAM, ADV, LdT)	44 (50.0)/44 (50.0)^b^

a *Continuous variables are expressed as median (IQR)*.

b *Categorical variables as number (percentage)*.

*HBV, Hepatitis B virus; ALT, alanine aminotransferase; HBeAg, hepatitis B e antigen; HBsAg, hepatitis B surface antigen; pgRNA, pregenome RNA; ETV, entecavir; LAM, lamivudine, ADV, adefovir dipivoxil; LDT, telbivudine*.

* *Means that the ULN of ALT is 50 IU/L for male and 40 IU/L for female*.

### Evaluation of the Treatment Effect During the 96-Month NAs Therapy

**Figure 1:**
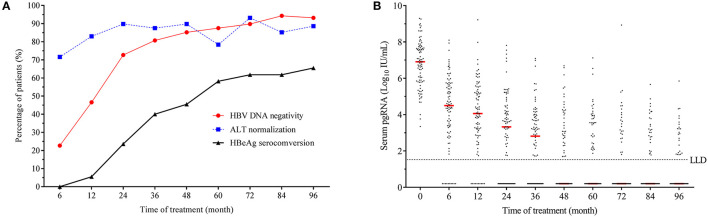
Dynamics of clinical parameters during the 96-month NAs therapy. **(A)** Dynamic change in the percentages of patients with HBV DNA negativity, ALT normalization and HBeAg seroconversion. **(B)** Dynamic change of serum pgRNA during the 96-month NAs treatment. LLD, lower limit of detection.

The dynamic changes in the percentages of patients with virological response, biochemical response and serological response during the whole process of antiviral therapy are recorded and shown in Figure 1A. Overall, the percentage of patients with ALT normalization varied slightly. The percentage of patients with undetectable HBV DNA increased rapidly from baseline to month 48 and then increased slowly afterwards. Approximately 84% (74/88) patients at month 48 and 94% (83/88) patients at month 96 achieved HBV DNA negativity. The percentage of patients with HBeAg seroconversion grew rapidly during the first 60-month therapy and then increased slowly. The median of serum pgRNA levels showed a sharp decline from baseline to month 48 and dropped lower the limit of the detection (LLD) at month 48 of treatment (Figure 1B). The decline of pgRNA in positive-HBeAg and negative-HBeAg patients showed a similar tendency (see Supplementary Figures 1A,B). Totally, 38 patients (27 positive-HBeAg and 11 negative-HBeAg) at year 4 and 24 patients (18 positive-HBeAg and 6 negative-HBeAg) at year 8 still had detectable serum pgRNA.

### Comparison of the Baseline Serum PgRNA Levels in Patients of Different Subgroups

**Figure 2:**
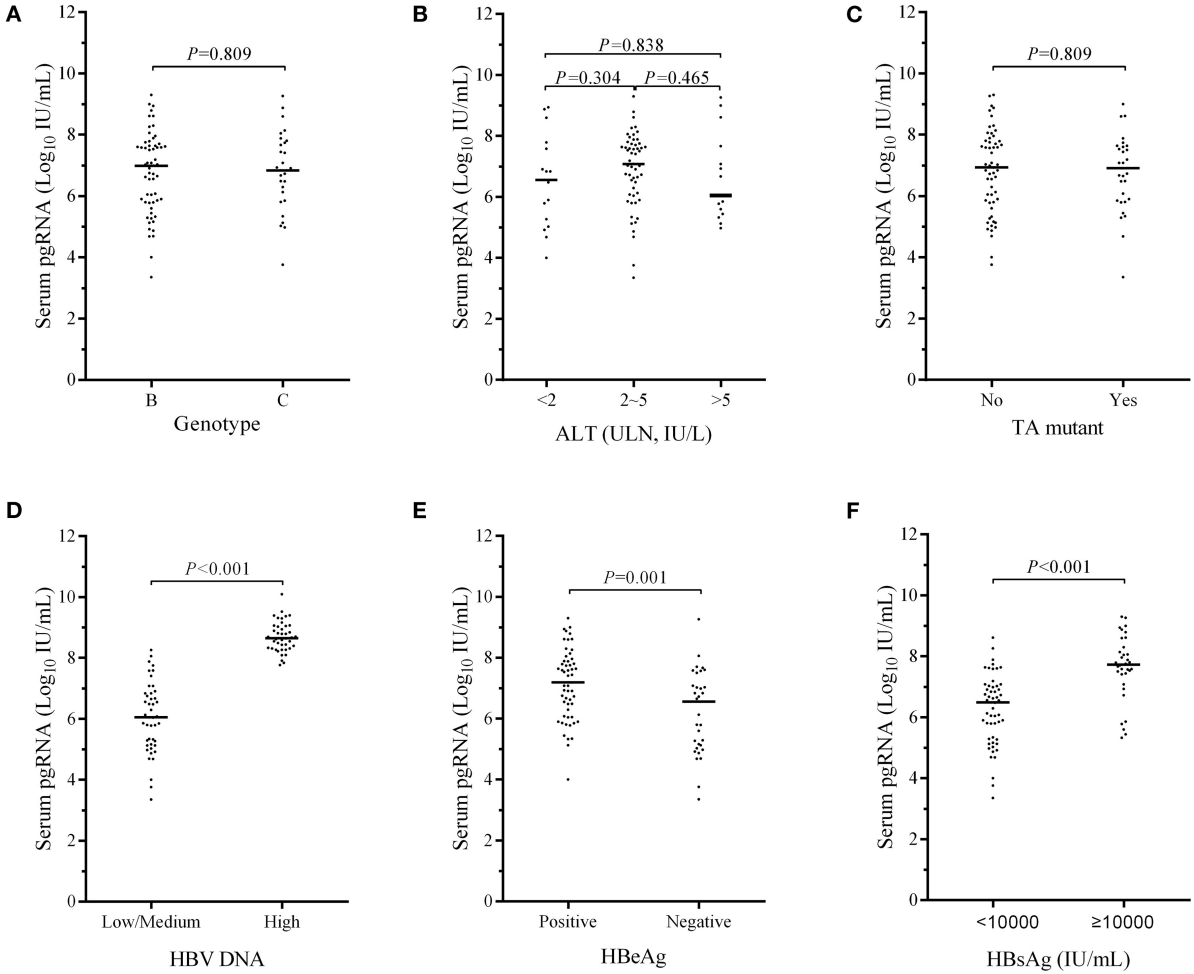
Comparison of the baseline serum pgRNA levels in CHB patients of different subgroups, that is, in patients of different genotypes **(A)**, different ALT levels **(B)**, with or without TA mutant **(C)**, low/medium and high HBV DNA levels **(D)**, positive- and negative- HBeAg **(E)**, and different HBsAg levels **(F)**. *P*-values between the groups were calculated using chi-squared test.

In the present study, patients with different ages (*p* = 0.597, see Supplementary Figure 1C) and sexes (*p* = 0.800, see Supplementary Figure 1D) showed no difference in serum pgRNA levels at baseline. The baseline serum pgRNA levels did not show any significant difference in patients with HBV genotype B and C (*p* = 0.809, Figure 2A), nor did it show any difference in patients with different ALT levels (Figure 2B). Likewise, no difference was observed in the baseline pgRNA levels between patients with or without TA mutant (*p* = 0.809, Figure 2C). Compared to patients with high viral loads, patients with low/medium HBV DNA levels had remarkable lower serum pgRNA levels at baseline (*p* < 0.001, Figure 2D). The baseline serum pgRNA levels were significant higher in HBeAg-positive than in HBeAg-negative patients (*p* = 0.001, Figure 2E). Statistical difference was also observed between patients with low (<10,000 IU/mL) and high serum HBsAg levels (>10,000 IU/mL) (*p* < 0.001, Figure 2F).

### Correlations Between Serum ALT, HBV DNA, HBsAg, and Serum PgRNA Levels at Baseline

**Figure 3:**
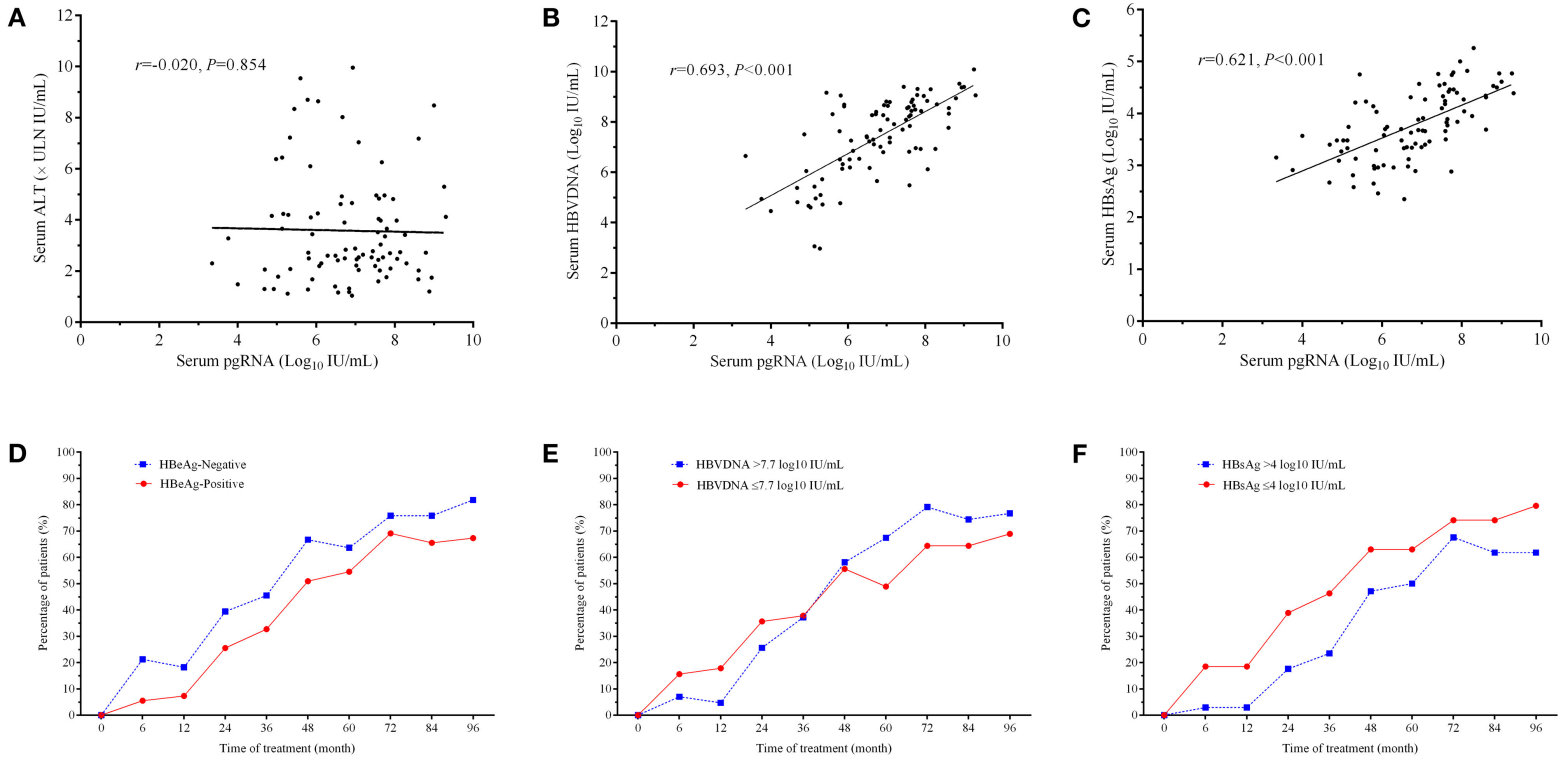
Correlations between serum ALT **(A)**, HBV DNA **(B)**, HBsAg levels **(C)**, and serum pgRNA levels at baseline. Dynamic changes in the percentages of patients with undetectable serum pgRNA during the 96-month NAs treatment in patients of positive- and negative- HBeAg **(D)**, in patients with either serum HBV DNA > 7.7 log10 IU/mL or ≤ 7.7 log10 IU/mL **(E)** and in patients with either HBsAg > 4 log10 IU/mL or ≤ 4 log10 IU/mL **(F)**. The correlation was tested by Spear-man's bivariate correlation.

The corrections between serum ALT, HBV DNA, HBsAg, and serum pgRNA levels at baseline are shown in Figures 3A–C. No correlation was observed between serum pgRNA and ALT levels (*r* = −0.020, *p* = 0.854, Figure 3A), whereas positive correlations were found between serum pgRNA levels and serum HBV DNA (*r* = 0.693, *p* < 0.001, Figure 3B), and HBsAg levels (*r* = 0.621, *p* < 0.001, Figure 3C).

### Dynamic Changes in the Percentages of Patients With Undetectable Serum PgRNA During the 96-Month NAs Therapy

The proportions of patients with undetectable serum pgRNA increased over time, irrespective of the HBeAg status, serum HBV DNA, and HBsAg levels (Figures 3D–F). The percentage of patients achieving undetectable serum pgRNA was always slightly higher in HBeAg-negative group (Figure 3D). Among patients with different levels of serum HBV DNA, higher percentage of patients with low/medium viral loads in the first 36-month therapy and higher percentage of patients with high viral loads (>7.7 log10 IU/mL) in the late 5 years of treatment achieved undetectable serum pgRNA (Figure 3E). Patients with low HBsAg levels ( ≤ 4 log10 IU/mL) showed higher response ratio (Figure 3F).

### Dynamic Changes of the Serum HBV DNA and PgRNA During the 96-Month NAs Therapy

**Figure 4:**
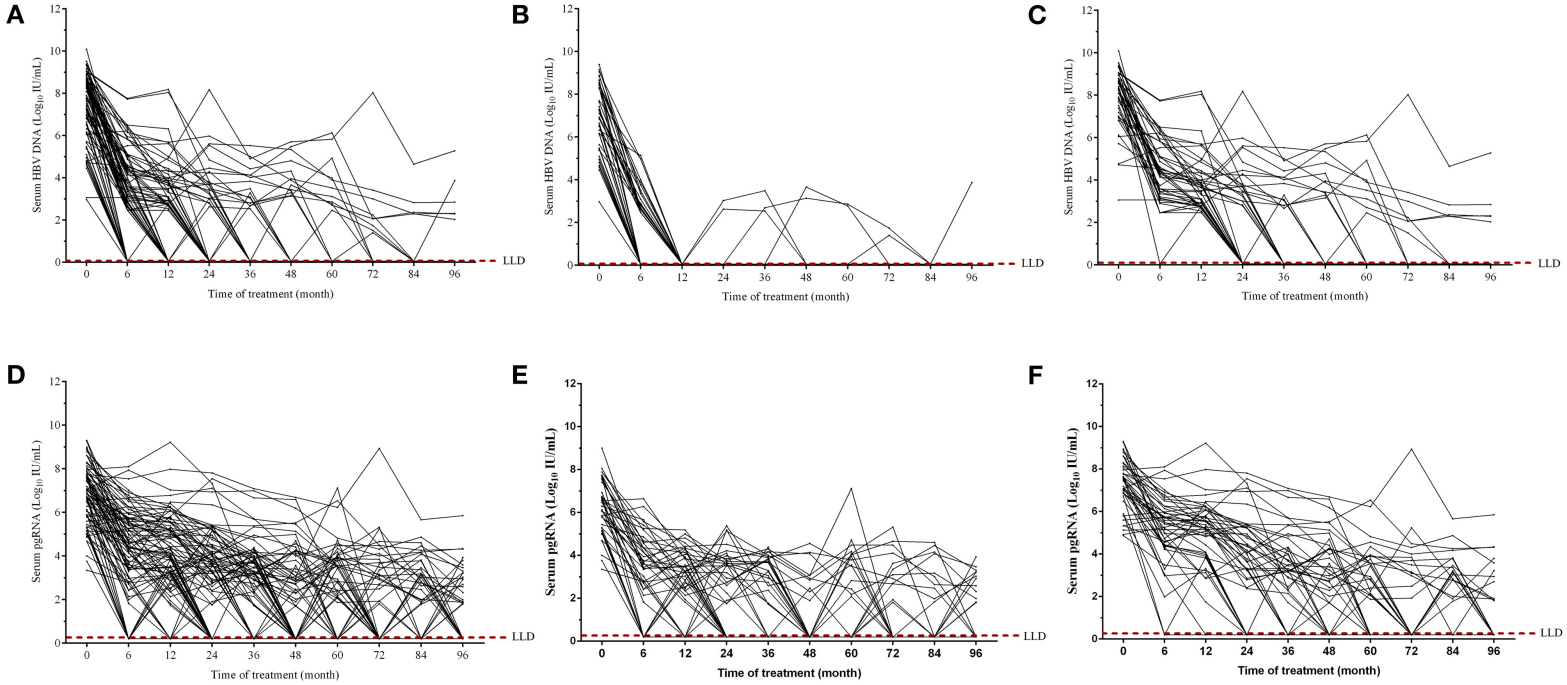
Dynamics of serum HBV DNA **(A–C)** and pgRNA **(D–F)** during the 96-month NAs treatment. Dynamic changes of serum HBV DNA and pgRNA levels from baseline to month 96 in all patients **(A,D)**, in patients treated with ETV **(B,E)**, and in patients treated with LAM/ADV/LdT treatment **(C,F)**.

Majority of the patients achieved virological response during the 96-month NAs antiviral therapy (Figure 4A). All patients treated with ETV had undetectable serum HBV DNA after half or 1 year therapy (Figure 4B). With the treatment continuation, 39 patients achieved sustained virological response, whereas other five individuals presented fluctuant serum HBV DNA, among which one patient occurred virological breakthrough at the end of our follow-up. For patients treated with non-ETV therapy (LAM/ADV/LdT), more than half of them achieved undetectable serum HBV DNA after 36-month therapy. However, four patients never achieved virological response throughout the whole process (Figure 4C).

Serum pgRNA levels were simultaneously monitored. During the 96-month NAs therapy, serum pgRNA levels were fluctuant and showed the tendency to descend. More and more patients achieved undetectable serum pgRNA over time (Figure 4D). Thirty-three patients receiving ETV therapy had undetectable serum pgRNA, while other 11 patients displayed detectable serum pgRNA levels at the end of our observation (about 2–4 log10 IU/mL) (Figure 4E). Thirteen patients receiving non-ETV therapy (LAM/ADV/LdT) had detectable serum pgRNA (about 2–6 log10 IU/mL) at the end of our follow-up (Figure 4F). Overall, the degree of serum HBV DNA decline was more pronounced than that of serum pgRNA. About 19 patients with negative HBV DNA showed detectable serum pgRNA after the 96-month NAs therapy.

### Clinical Indicators Predicting Undetectable Serum PgRNA After 4-Year NAs Therapy

In the present study, the median of serum pgRNA levels dropped lower the limit of the detection at year 4 after NAs therapy. Clinical indicators predicting undetectable serum pgRNA at year 4 were calculated with univariable and multivariate logistic regression analysis. As shown in Table 2, TA mutant (*p* = 0.017), serum HBsAg (*p* = 0.034), baseline serum pgRNA (*p* = 0.015), and serum pgRNA decline at month 6 (*p* = 0.044) were significantly associated with undetectable serum pgRNA at year 4. Further multivariate regression analysis revealed that baseline serum pgRNA (*p* = 0.022) and pgRNA decline at month 6 (*p* = 0.044) were independent predictors for undetectable serum pgRNA at year 4 after NAs therapy (Table 3). Serum pgRNA of low level at baseline or great decline at month 6 may predict high incidence of undetectable serum pgRNA at year 4 following NAs therapy.

**Table 2:** Univariable logistic regression analysis of factors associated with undetectable level of serum pgRNA at year 4.

**Parameter**	**4-year**			**8-year**		
	**OR**	**95% CI**	***P*-value**	**OR**	**95% CI**	***P*-value**
Age	0.960	0.906–1.017	0.168	0.973	0.914–1.035	0.384
Gender (ref: female)	1.314	0.518–3.333	0.566	1.382	0.502–3.810	0.531
Family history of hepatitis B (ref: yes)	1.714	0.725–4.056	0.220	2.429	0.887–6.651	0.084
ALT	1.075	0.879–1.316	0.480	0.899	0.728–1.109	0.320
HBeAg (ref: negative)	0.519	0.212–1.271	0.151	0.457	0.160–1.304	0.143
Genotype (ref: B)	1.381	0.557–3.424	0.486	0.168	0.586–4.836	0.334
TA mutant (ref: wildtype)	3.194	1.226–8.325	0.017	1.923	0.672–5.504	0.223
Serum HBV DNA	0.951	0.725–1.248	0.719	0.977	0.722–1.322	0.881
Serum HBsAg	0.480	0.244–0.946	0.034	0.627	0.303–1.297	0.208
Antiviral treatment (ref: other)	1.204	0.517–2.801	0.667	1.258	0.491–3.223	0.632
Baseline pgRNA	0.639	0.445–0.918	0.015	0.755	0.515–1.108	0.151
pgRNAdecline at 6-month	1.302	1.007–1.682	0.044	1.069	0.818–1.397	0.625

*Associations were evaluated by univariable logistic regression. Odds ratio (OR) and 95% confidence interval (CI) were calculated*.

*P-value <0.05 was considered statistically significant*.

*HBsAg, hepatitis B surface antigen; pgRNA, pregenome RNA*.

**Table 3:** Multivariate regression analysis of factors associated with undetectable level of serum pgRNA at year 4.

**Parameter**	**OR**	**OR 95% CI**		***P*-value**
		**Low**	**Up**	
TA mut	2.359	0.805	6.912	0.118
HBsAg	1.050	0.415	2.655	0.919
HBVRNA	0.551	0.332	0.916	0.022
pgRNA decline at 6-month	1.385	1.008	1.902	0.044

*HBsAg, hepatitis B surface antigen; pgRNA, pregenome RNA; OR, Odds ratio; CI, confidence interval*.

### Relationships Between HBeAg Seroconversion, HBsAg Levels, and the Baseline Serum PgRNA Levels

**Figure 5:**
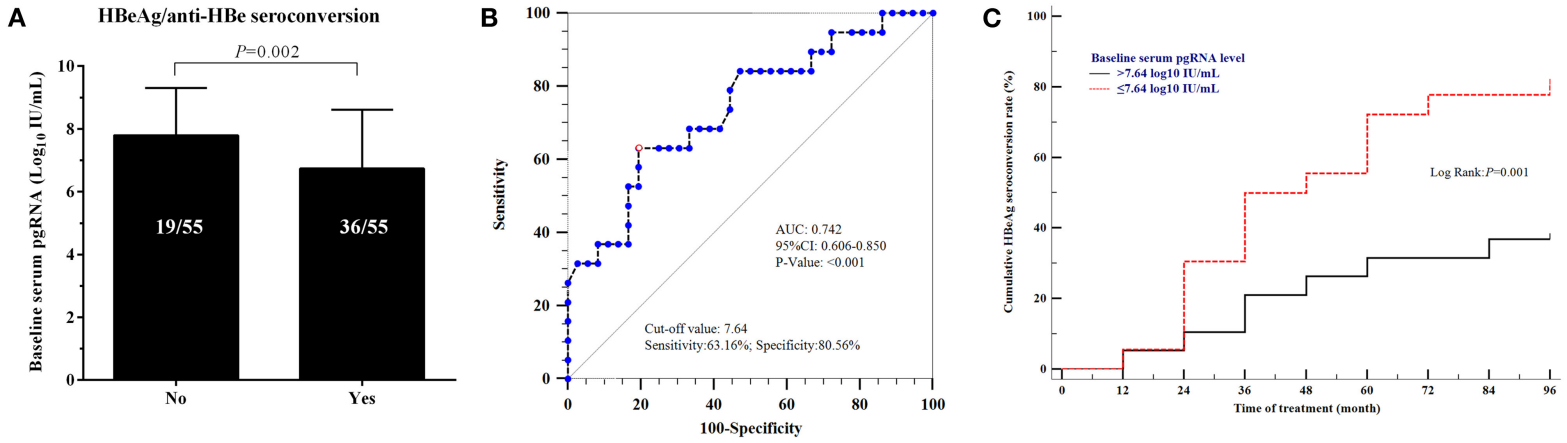
Relationships between HBeAg seroconversion and baseline serum pgRNA levels. **(A)** Comparison of the levels of baseline serum pgRNA in patients with/without HBeAg seroconversion. Data were compared using *t-*test. **(B)** Predictive value of baseline serum pgRNA for HBeAg seroconversion using ROC curve analysis. **(C)** Cumulative rate of HBeAg seroconversion in patients with baseline serum pgRNA >7.64 log10 IU/mL and ≤ 7.64 log10 IU/mL.

We further explored the relationships between HBeAg seroconversion, HBsAg levels and the baseline serum pgRNA levels. Thirty-six patients with HBeAg seroconversion had lower baseline serum pgRNA levels than those without seroconversion (6.793 ± 0.182 vs. 7.758 ± 0.239, *p* = 0.002, Figure 5A). The predictive value of baseline serum pgRNA for HBeAg seroconversion was calculated with ROC. The area under the curve (AUC) of baseline serum pgRNA was 0.742 (95% CI: 0.606–0.850) with 63.16% sensitivity and 80.56% specificity (Figure 5B). Moreover, the cumulative rate of HBeAg seroconversion was significant higher in patients with baseline serum pgRNA ≤ 7.64 log10 IU/mL compared to those with baseline serum pgRNA > 7.64 log10 IU/mL (Figure 5C), indicating that baseline serum pgRNA may be a potential biomarker for predicting HBeAg seroconversion in patients with regular long-term NAs therapy.

However, the baseline serum pgRNA may not be associated with the change of HBsAg levels. At the end of our observation, total of 83 patients achieved negative HBV DNA. Among them, 56 patients still present with HBsAg>500 IU/mL and 27 patients ended with HBsAg <500 IU/mL, and the baseline serum pgRNA levels were not statistically different between these patients (6.558 ± 0.293 vs. 6.996 ± 0.156, *p* = 0.152, see Supplementary Figure 2A). Among 64 patients with both negative HBV DNA and RNA, the baseline serum pgRNA levels also showed no significant difference between patients of HBsAg > 500 IU/mL and HBsAg <500 IU/mL (6.272 ± 0.360 vs. 6.868 ± 0.164, *p* = 0.087, see Supplementary Figure 2B).

## Discussion

NAs strongly suppress viral replication and decrease serum HBV DNA to undetectable level *via* inhibiting the viral polymerase, but they cannot prevent the formation of cccDNA from incoming virions and the subsequent expression of HBV genes from cccDNA (15). As a surrogate marker for the transcriptional activity of cccDNA in the liver, serum pgRNA has attracted increasing attention in recent years. This study analyzed the kinetics of serum pgRNA in chronically HBV-infected patients and the correlations between serum pgRNA and other common clinical indicators, aiming to probe the potential role of serum pgRNA in evaluating the antiviral efficacy in patients who received 96-month NAs therapy.

In the present study, levels of serum HBV DNA and pgRNA decreased as the treatment time prolonged. Given that serum HBV pgRNA is transcribed from intrahepatic cccDNA, while NAs inhibit HBV replication and have no direct effect on cccDNA and cccDNA-derived viral transcripts, we herein found serum HBV DNA decreased more quickly than serum pgRNA both in ETV-treated and non-ETV-treated patients, in accordance with a previous report (16). It seems that the influence of NAs therapy on serum pgRNA level is limited, but may be amplified over time. We monitored the dynamic change of serum pgRNA during the long-term NAs therapy, and found that serum pgRNA in most patients, generally speaking, showed a decreasing trend from baseline to 96 months after treatment, which was contrast with the earlier results that serum pgRNA increased both in CHB patients (10) and in HBV transgenic mice with NAs therapy (9). The reason may be that NAs can block the HBV polymerase-mediated synthesis of rcDNA from the pgRNA template, resulting in the excessive accumulation of encapsidated pgRNA during a short treatment period (17). However, along with the time of treatment, NAs might eventually, to some extent, reduce the replenishment of cccDNA pool *via* the suppression of rcDNA, thereby inhibiting the generation of HBV RNA virions.

Serum pgRNA levels were found still positive and detectable in some patients with undetectable serum HBV DNA in this study, in agreement with the previous studies that pgRNA might be a new biomarker of different significance from that of HBV DNA during NAs treatment (18, 19). Currently, achieving virological reponse, meaning sustained serum HBV DNA below the lower limit of detection, is one of the prerequisites for discontinuation of NAs therapy. In the present study, the limit of the detection of HBV DNA was set for 100 IU/mL, and we observed positive pgRNA in patients with negative HBV DNA, and other investigators also proved that pgRNA could be detected even when HBV DNA was below 20 IU/mL (20). Patients with sustained negative HBV DNA but positive serum pgRNA experienced viral rebound at high frequency after discontinuation of NAs therapy (9). All the foregoing demonstrated that serum pgRNA might be more accurate than serum HBV DNA in evaluating the endpoint of antiviral drugs in patients with CHB. Hence, a redfined virological response (persistent loss of serum HBV DNA and pgRNA) has been proposed (21). Compared with serum HBV DNA or pgRNA alone, total serum nucleic acids (HBV DNA plus pgRNA) may better reflect the activity of intrahepatic cccDNA and may be more valuable to guide therapy discontinuation (21–23).

The relationships between serum HBV DNA, HBsAg, and serum pgRNA levels at baseline were then evaluated. Previous studies suggested that the serum pgRNA levels were correlated with HBV DNA (24) and HBsAg (25) before treatment. Both Liu et al. (26) and Jansen et al. (16) reported that serum pgRNA levels were positively correlated with HBV DNA and HBsAg before treatment in HBeAg-positive patients, whereas in HBeAg-negative patients, serum pgRNA was only correlated with serum HBV DNA. In the present study, we not only observed that the levels of serum pgRNA were statistically different in patients with different levels of serum HBV DNA and HBsAg at baseline, but also found the strongly positive correlations between serum HBV DNA, HBsAg, and serum pgRNA levels at baseline. However, we did not further distinguish their correlations in either HBeAg-positive or -negative patients.

Serum HBsAg loss is widely accepted as the treatment endpoint. However, no patients achieved the goal of “functional cure” and no patients discontinued antiviral therapy in the present study, and we thus did not compare the potential between serum pgRNA and HBsAg in predicting drug withdrawal. Although positive correlation was found between baseline serum pgRNA and HBsAg levels, serum pgRNA levels were not significantly different between patients with different levels of HBsAg after NAs treatment, demonstrating that the baseline serum pgRNA may be not an optimal indicator for predicting HBsAg seroconversion in patients treated with NAs. In contrast, patients with or without HBeAg seroconversion showed different baseline serum pgRNA, and the baseline serum pgRNA appeared to serve as a predictor for HBeAg seroconversion with relative high sensitivity and specificity in HBeAg-positive patients, which was strongly supported by another study in which patients were treated with ETV (27). Thus, patients with low serum pgRNA levels at baseline may be more likely to achieve HBeAg seroconversion.

TA mutant, or termed A1762T and G1764A exchange present in the HBV genome basal core promoter (BCP) region, is a common HBV variant during long-term NAs therapy (28). HBV strain with TA mutant increased the levels of pgRNA transcription and HBV DNA replication (29), thus it was expected that TA mutant was a risk factor for undetectable serum pgRNA. In the present study TA mutant indeed seemed to be negatively associated with undetectable serum pgRNA at year 4 after treatment using univariable logistic regression analysis. Further multivariate logistic regression analysis revealed that baseline serum pgRNA levels and pgRNA decline levels at month 6 were considered as independent predictors for undetectable level of serum pgRNA at year 4 post treatment. Patients with low level of serum pgRNA at baseline or great decline of serum pgRNA at month 6 may have a great chance to reach the goal of undetectable serum pgRNA at year 4.

The main limitation in the study is that we have not detected the intrahepatic cccDNA levels and cannot dynamically observe the dynamic change of intrahepatic cccDNA during the whole process of antiviral therapy, and we therefore cannot investigate the correlations between serum HBV DNA, HBsAg, HBeAg, pgRNA, and intrahepatic cccDNA levels before and after treatment.

In conclusion, serum pgRNA showed dynamic change during long-term NAs therapy. Serum pgRNA of low level at baseline or great decline at month 6 may predict the high incidence of undetectable serum pgRNA at year 4 following NAs therapy, and the baseline serum pgRNA may serve as a novel predictor for HBeAg seroconversion during NAs therapy.

## Data Availability Statement

The original contributions presented in the study are included in the article/Supplementary Materials, further inquiries can be directed to the corresponding author/s.

## Ethics Statement

The studies involving human participants were reviewed and approved by Ethics Committee of West China Hospital of Sichuan University. Written informed consent for participation was not required for this study in accordance with the national legislation and the institutional requirements.

## Author Contributions

EC and HT contributed to the study concept and design. YT and MW contributed to the accuracy of the data analyses and manuscript drafting. JL, XC, MH, DZ, and TZ contributed to the acquisition of data. All authors contributed to the analysis and interpretation of data and approved the final version of the manuscript.

## Funding

This work was supported by grants from National Thirteen-Five Project of China (2017ZX10302201-006-003).

## Conflict of Interest

JC is employed by Shanghai RenDu Biotechnology Co. Ltd. The remaining authors declare that the research was conducted in the absence of any commercial or financial relationships that could be construed as a potential conflict of interest.

## Publisher's Note

All claims expressed in this article are solely those of the authors and do not necessarily represent those of their affiliated organizations, or those of the publisher, the editors and the reviewers. Any product that may be evaluated in this article, or claim that may be made by its manufacturer, is not guaranteed or endorsed by the publisher.

## Supplementary Material

Supplementary Figure 1 Dynamic changes of serum pgRNA in HBeAg-positive **(A)** and -negative **(B)** patients. Comparison of the baseline serum pgRNA levels in patients of different age **(C)** and different sex **(D)**. *P*-values between the groups were calculated using chi-squared test.

Supplementary Figure 2 Among patients with negative HBV DNA **(A)**, and with negative HBV DNA and pgRNA **(B)** at month 96, baseline serum pgRNA levels were compared between the groups of HBsAg <500 IU/mL and > 500 IU/mL. *P*-values between the groups were calculated using chi-squared test.
