# Supplementary figures and images for: Dynamics of Serum Pregenome RNA in Chronic Hepatitis B Patients Receiving 96-Month Nucleos(t)ide Analog Therapy
Source: Front Med (Lausanne). 2022 Feb 28;9:787770. doi: 10.3389/fmed.2022.787770 (PMC8918695; doi:10.3389/fmed.2022.787770)

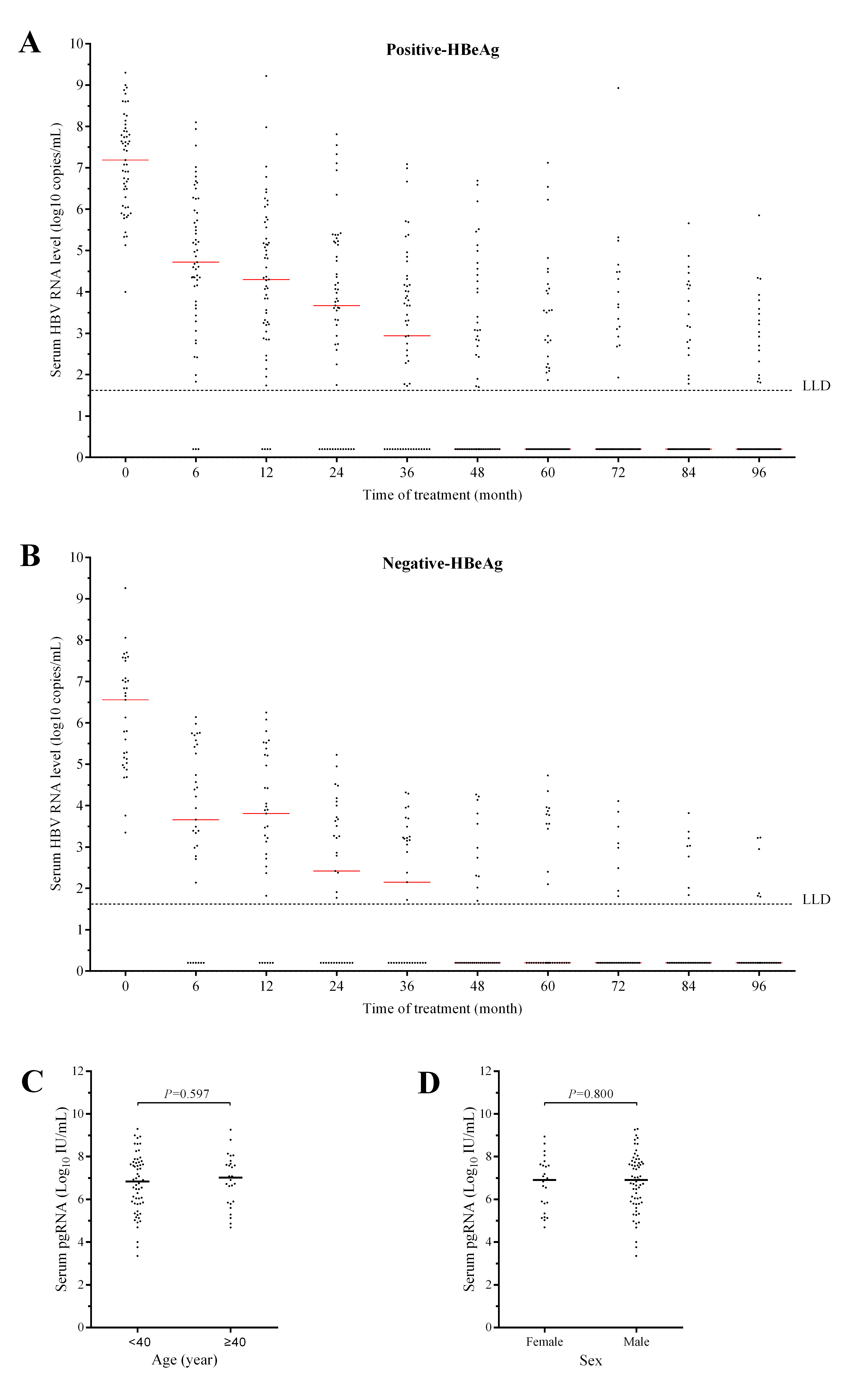

Supplement: Supplementary Figure 1 — Dynamic changes of serum pgRNA in HBeAg-positive (A) and -negative (B) patients. Comparison of the baseline serum pgRNA levels in patients of different age (C) and different sex (D). P-values between the groups were calculated using chi-squared test. [file Image_1.TIF]

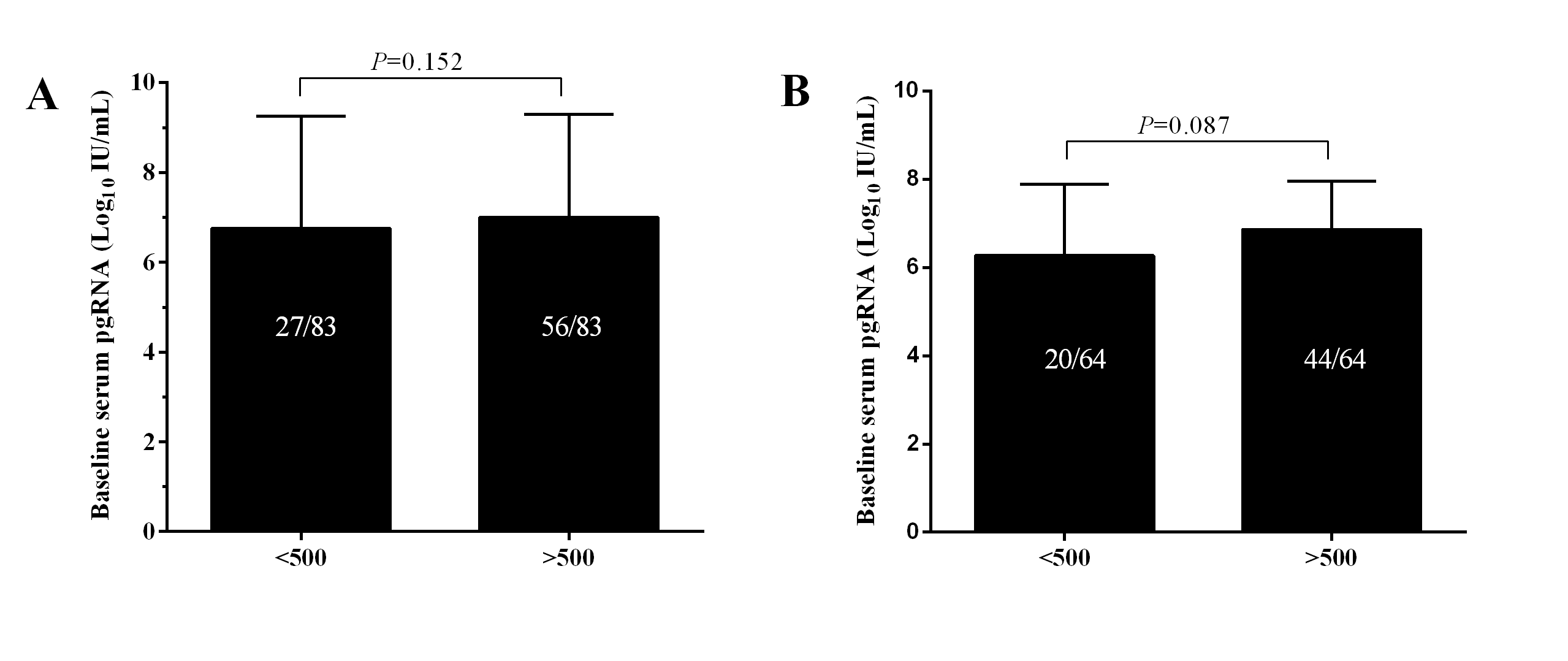

Supplement: Supplementary Figure 2 — Among patients with negative HBV DNA (A), and with negative HBV DNA and pgRNA (B) at month 96, baseline serum pgRNA levels were compared between the groups of HBsAg <500 IU/mL and > 500 IU/mL. P-values between the groups were calculated using chi-squared test. [file Image_2.TIF]
